# Changes in nasal air flow and school grades after rapid maxillary expansion in oral breathing children

**DOI:** 10.4317/medoral.17810

**Published:** 2012-02-09

**Authors:** Hilda Torre, Jose A. Alarcón

**Affiliations:** 1 PhD in Dentistry. Department of Stomatology, Area of Orthodontics, School of Dentistry, University of Granada, Granada, Spain; 2 PhD in Dentistry. MS in Orthodonctics. Associate Professor. Department of Stomatology, Area of Orthodontics, School of Dentistry, University of Granada, Granada, Spain; 3 ….

## Abstract

Objective: To analyse the changes in nasal air flow and school grades after rapid maxillary expansion (RME) in oral breathing children with maxillary constriction. Material and Methods: Forty-four oral breathing children (mean age 10.57 y) underwent orthodontic RME with a Hyrax screw. Forty-four age-matched children (mean age 10.64 y) with nasal physiological breathing and adequate transverse maxillary dimensions served as the control group. The maxillary widths, nasal air flow assessed via peak nasal inspiratory flow (PNIF), and school grades were recorded at baseline, and 6 months and one year following RME. Results: After RME, there were significant increases in all the maxillary widths in the study group. PNIF was reduced in the study group (60.91 ± 13.13 l/min) compared to the control group (94.50 ± 9.89 l/min) (P < 0.000) at the beginning of the study. Six months after RME, a significant improvement of PNIF was observed in the study group (36.43 ± 22.61). School grades were lower in the study group (85.52 ± 5.74) than in the control group (89.77 ± 4.44) (P < 0.05) at the baseline, but it increased six months after RME (2.77 ± 3.90) (P < 0.001) and one year later (5.02 ± 15.23) (P < 0.05). Conclusions: Nasal air flow improved in oral breathing children six months and one year after RME. School grades also improved, but not high enough to be academically significant.

** Key words:**Maxillary constriction, oral breathing, nasal air flow, rapid maxillary expansion, school grades.

## Introduction

Oral breathing is considered a sign of insufficient nasal air flow. Children who breathe orally generally have an increased frequency of sleep disorders, obstructive sleep apnea, sleep-related breathing disorders, restlessness, excessive daytime sleepiness, lack of attention, behavioral and neurocognitive abnormalities and decreased school performance (1,2). Most studies of malocclusions in children who breathe orally show that they have a high prevalence of maxillary constriction and accompanying posterior crossbite (3,4). A positive correlation (5) and, in some cases, even a direct cause–effect relationship between mouth-breathing and posterior cross bite has been described (6,7).

To quantify the degree of nasal obstruction, techniques such as rhinomanometry, acoustic rhinometry and active anterior rhinomanometry are employed; however, these methods require complex, expensive equipment and highly trained operators (8). Peak nasal inspiratory flow (PNIF) has been shown to be a sensitive method for assessing nasal patency, and its advantage is that it can be used in orthodontic clinics after a short training period (8-10).

Rapid maxillary expansion (RME) is the treatment of choice for the correction of maxillary constriction accompanied by a uni- or bilateral posterior cross bite. Following RME, studies have reported significant decreases in nasal airway resistance (11-14) and subjective improvement in nasal respiration (14). Nevertheless, the clinical significance of these findings is questionable , and scientific investigations producing evidence-based data are scarce (1,15).

The aims of this study were to analyse the changes in nasal air flow and school grades after RME in oral breathing children with maxillary constriction.

## Material and Methods

Patient population

Forty-four oral breathing children (22 boys and 22 girls, mean age 10.57 ± 1.93 y) were consecutively recruited from referrals to the Pediatric Clinic at the School of Dentistry where the study took place. The children in this study group had a history of oral breathing, confirmed by their parents and the medical history. On clinical examination these patients showed lip inefficiency at rest, dental crowding in the upper arch, ‘‘adenoidal facies’’ and transverse maxillary constriction, accompanied by a uni- or bilateral posterior cross bite. Evaluation of the breathing pattern showed a diaphragmatic mode of inhalation with under expansion of the thorax and a reduced mobility of the nostrils suggesting a reduced patency of the upper airway. Oral breathing was shown by water vapor condensed on the surface of a mirror placed outside the mouth. Forty-four children (22 boys and 22 girls, mean age 10.64 ± 1.64 y), matched by age and gender, who displayed physiological nasal breathing and adequate transverse dimensions of the maxilla, were consecutively recruited from the same clinic to serve as the control group.

All children involved in the study were of similar origin, had similar socioeconomic and cultural conditions and came from the same residential area. The exclusion criteria for both groups were cleft lip and palate or craniofacial anomalies, previous or current orthodontic treatment, chronic medical illness causing frequent absences from school of more than 5 days in a term and a very poor socioeconomic status with reliance on monthly welfare support, that could affect the children’s school grades (16).

All patients’ parents were informed on the characteristics of the study and agreed to participate by signing an EC-approved informed consent.

Therapy

The children in the study group were treated with a Hyrax palatal expander (Dentaurum®, Germany) as the only treatment. All appliances were manufactured, cemented and activated by the same operator (HT) according to the following protocol: following an initial activation of two-fourth turns (0.4 mm), the parents were instructed to activate the screw one-fourth turn (0.2 mm) twice per day, until overcorrection of the transverse relationship of 3 mm was seen (on average 18 ± 2 days). Patients were monitored weekly. The palatal expander was then stabilized and kept in situ for retention for 6 months.

Dental study

Alginate impressions were taken for all of the children. The impressions were poured on the same day with hard dental stone. For each patient in both groups, study models were taken before treatment (T0), six months after the end of RME (T1), and 1 year later (T2). Measurements were made directly on the maxillary dental casts with an electronic digital caliper (Mitutoyo, Mitutoyo Corporation, Aurora, IL, USA) and recorded to an accuracy of 0.01 mm. The landmarks used for the width measurements were as follows: maxillary inter canine widths (cusp tips of the canines), maxillary inter-first premolar widths (distal pits of the maxillary first premolars), maxillary inter-second premolar widths (distal pits of the maxillary second premolars) and maxillary inter molar widths (central fossae of the maxillary first molars).

Twenty study models were selected randomly, and arch width measurements were performed twice on two separate occasions with an interval of two weeks. The intra-observer error was assessed as recommended by Dahlberg and Houston (17).

Nasal air flow evaluation

Nasal air flow was measured by PNIF (l/min) using an In-Check portable nasal inspiratory flow meter (Clement Clarke International, Harlow, Essex, UK). PNIF was measured at T0, T1 and T2. During the experiment, each subject was seated in a dental chair in an upright position with the Fränkfurt plane parallel to the floor in a quiet, dark and comfortable environment. All subjects received appropriate instructions on how to use the PNIF meter correctly prior to the measurements and were supervised while the readings were obtained. The children were asked to inhale in the flow meter mask as deeply as possible. Three measurements were recorded for each patient. Since a ‘training effect’ (8) may occur, only the second and third readings were used to determine the measurement repeatability, and the higher of these two values was used. One trained examiner performed all of the measurements without knowledge of the presence/absence of maxillary constriction, and the patient data remained blinded throughout the analysis.

School grades

The grades (expressed as numbers) were obtained for each of the subjects that comprised the curriculum. Data were extracted from bulletins provided by the children’s parents, and an arithmetic average of the grades for all subjects was obtained to provide a single value for each child. Data were collected at T0, T1 and T2.

Statistical analysis

Means, standard deviations and 95% confidence intervals (CIs) were calculated for all variables. The t-test for paired samples was applied to compare the means between the three time points in the study (T1-T0, T2-T1 and T0-T2) in each group for every variable. The Student’s t-test was used to compare differences between the groups. Significance was set at the 5% level (P ≤ 0.05). In the study group, the Pearson correlation coefficient (r) (α = 0.05) was used to determine associations between changes in the dental variables, PNIF and school grades. All statistical analyses were performed using SPSS 16.0 software (SPSS Inc., Chicago, IL, USA).

## Results

Dental variables

The mean intra-observer error ranged from 0.25 to 0.29 mm. The coefficient of reliability ranged from 95 to 99%. These findings indicate that the errors were minimal and unlikely to bias the results.

( Table 1) shows the mean values and standard deviations of the dental variables recorded in the maxillary arch. T-tests showed that there were statistically significant differences in all of the dental variables between the two groups (P < 0.000), reflecting smaller inter canine (4.45 ± 2.93 mm), inter-first premolar (7.78 ± 4.48 mm), inter-second premolar (6.30 ± 4.50 mm) and inter-first molar (6.34 ± 4.34 mm) widths in the study group children. Six months after RME, there was a significant increase in all the maxillary widths measured in the study group in comparison to the control group (T1-T0), and this increase was maintained one year later (T2-T0).

**Table 1:**
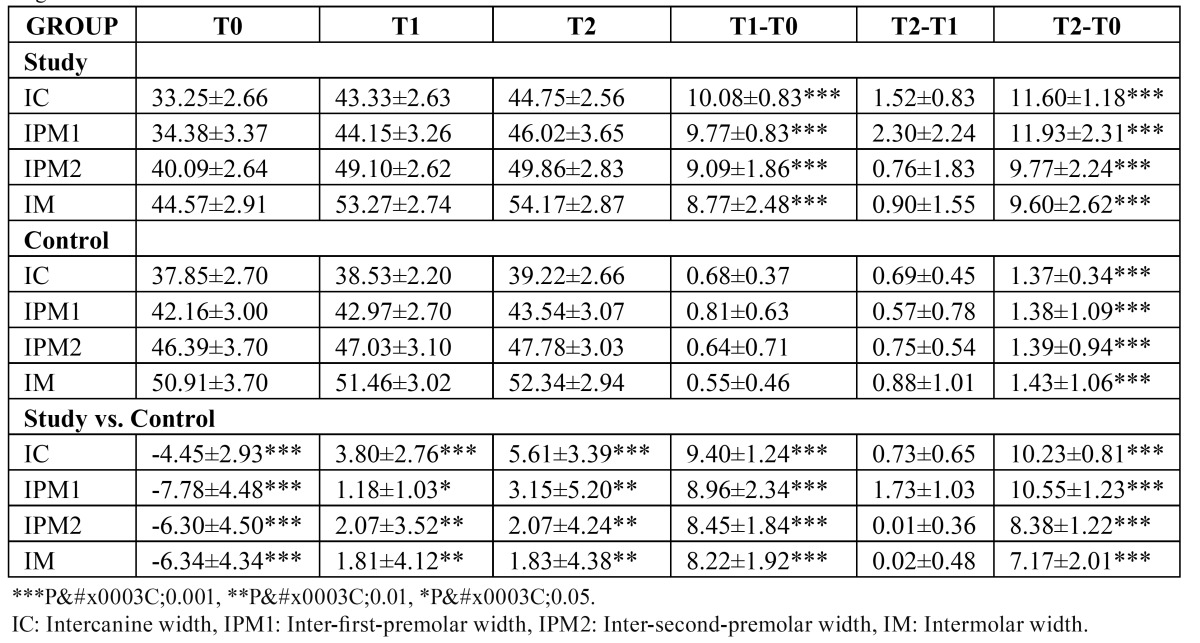
Means for the dental variables and differences between means in the study and in the control groups at the three stages.

Nasal air flow

The intra-class correlation coefficient was 0.91, and the 95% limits of agreement were ±27 l/min, showing that the PNIF measurements were reproducible.

Oral breathing children exhibited significantly lower nasal air flow (60.91 ± 13.13 l/min) than the control children (94.50 ± 9.89 l/min) at the beginning of the study (T0) (P < 0.000). Six months after RME, a significant improvement in PNIF was observed in the study group (36.43 ± 22.61) in reference to the control group (1.64 ± 4.68). At T1 and one year later (T2) there were no differences in PNIF between the two groups ( Table 2).

**Table 2:**
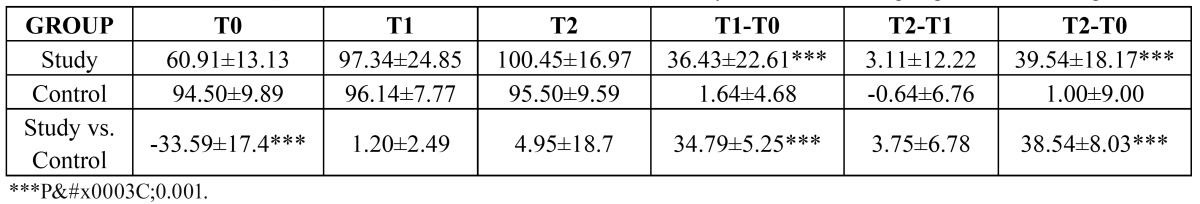
Means for the PNIF (l/min) and differences between means in the study and in the control groups at the three stages.

School grades

Before treatment, the average school grades were significantly lower in the study group (85.52 ± 5.74) than in the control group (89.77 ± 4.44) (P < 0.05) ( Table 3). In the study group, school grades increased six months after RME (2.77 ± 3.90) (P < 0.001) and one year later (5.02 ± 15.23) (P < 0.05). The difference between the groups from the beginning of the study to one year later (T2-T0) was also significant (5.54 ± 3.00). Pearson correlation showed a weak association between PNIF and school grades changes during the T2-T0 period (r = 0.38, P < 0.05).

**Table 3:**
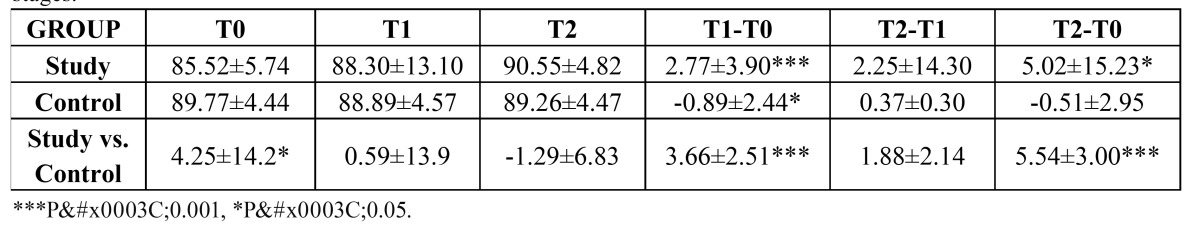
Means for the school grades and differences between means in the study and in the control groups at the three stages.

## Discussion

In this study, we measured nasal air flow and school grades in a group of oral breathing children with maxillary constriction and posterior cross bite before and after RME treatment to elucidate the possible relationships among maxillary constriction, nasal air flow deficiency and school grades. The children affected by this malocclusion had significantly lower dental-traverse maxillary dimensions, nasal air flow rates and school grades compared with the control children. Six months after the end of RME treatment, all the variables increased in the treated children. These results remained stable one year later, demonstrating the advantages of RME in children with these problems.

At the beginning of the study, all of the dental measurements (inter canine, inter-first premolar, inter-second premolar and inter-first molar widths) were significantly reduced in the study group. These results were expected, as only oral breathing children with maxillary constriction and posterior cross bite were included in the study. Six months after treatment with the Hyrax expander, all maxillary dental widths increased significantly and remained stable one year later. The expansion of the inter canine width was greater than that in the inter molar area. These results confirm the efficacy of the RME to manage maxillary transverse deficiencies as reported previously (18-20).

In this study, PNIF was the method chosen to investigate nasal air flow, since it is a simple, noninvasive and objective method. It has been used in several stu-dies to assess nasal patency, where it was shown to be as sensitive as acoustic rhinometry and active anterior rhinomanometry (8-10,21). Another advantage of this method is that it does not alter the patient’s behaviour because there are no attachments that enter the nose, as in other techniques. The disadvantage of this method is that the In-Check portable nasal inspiratory flow meter can give inaccurate results if the patient is positioned incorrectly. To prevent errors during this study, a standardized patient position was applied.

Oral breathing children showed significantly lower nasal air flow than control children. In other studies, reduced transverse maxillary dimensions have been associated with higher nasal airway resistance (15,22,23). Reduced nasal air flow affects normal nasal breathing patterns, and can result in oral breathing. A high frequency of maxillary constriction and posterior cross bite has been reported in oral breathers previously (5,23,24). The results of our study agree with these findings and suggest that oral breathing children with maxillary constriction require a multidisciplinary clinical approach involving orthodontists, pediatricians, otorhinolaryngologists and allergists.

After RME, there was a highly significantly increase in PNIF in children with maxillary constriction compared with controls, and there was no longer a difference between the two groups, reflecting the benefits of RME in these patients. This is a remarkable finding, since one of the most desirable effects of RME in oral breathing children with maxillary constriction is the improvement of nasal air flow. This improvement is probably due to an increase in nasal volume or area (11-13,25,26) and a decrease in nasal airway resistance (11-13,22,27,28), which probably results in better nasal breathing after RME (11,14,27). Nevertheless, it is difficult to compare our results with those obtained by others authors because of the heterogeneity of study designs, types of palatal expanders used, patients ages and methods employed to explore nasal air flow.

School grades were extracted from academic bulletins, following the recommendations of education researchers (29). Nevertheless, only examining school grades provided by parents may not necessarily provide a global assessment of “school performance”. Furthermore, there are many variables involved in school academic performance that must be considered, such as IQ, personality, social skills, parental and teacher expectations, socioeconomic conditions, parents’ education levels, motivation, self-control, the number of students in the class, teaching materials and school type (public or private). Other abnormalities, such as obstructive sleep apnea or sleep-related breathing disorders, may also cause poor school grades.

At the beginning of the study, the school grades were lower in oral breathing children than in the control children. Six months after treatment and one year later, no differences were observed between the groups. School grades increased by 2.77 ± 3.90 six months after RME and by 5.54 ± 3.00 one year later. Finally a weak positive correlation was observed between changes in PNIF and changes in school grades during the T2-T0 period. These results could suggest that RME increases nasal air flow, which could improve nasal breathing, and therefore improve the development of daily activities, including school performance. This improvement in school grades may also be secondary to treatment of sleep disordered breathing which coincided with the improvement of the oral breathing. Nevertheless, the increment in the school grades is neglect able level, so the clinical significance of this finding is questionable. On the other hand, although an association was shown between PNIF and school grades increments that does not imply a cause and effect relationship with the RME. Future studies controlling all the factors which could have an influence on the school performance, sleep quality and breathing problems are needed to achieve evidence-based data.
